# Meniscal Allograft Transplantation With Hybrid Fixation to Optimize Graft Tension: A Surgical Technique

**DOI:** 10.1016/j.eats.2025.103818

**Published:** 2025-08-29

**Authors:** Benjamin Trigg, Helena Franco, Francois Tudor

**Affiliations:** a Department of Orthopaedic Surgery, Gold Coast University Hospital, Southport QLD, Australia; b Faculty of Health Sciences and Medicine, Bond University, Robina QLD, Australia; c Griffith School of Medicine and Dentistry, Southport QLD, Australia

## Abstract

Meniscal allograft transplantation is an established procedure for restoring knee joint biomechanics and potentially delaying osteoarthritis progression in symptomatic, meniscus-deficient patients. This Technical Note describes an arthroscopic meniscal allograft transplantation approach using a posterior root bone plug and anterior root soft tissue fixation. This hybrid fixation technique is designed to produce stable root fixation while allowing adjustment of the graft position to accommodate minor graft/recipient size mismatch. The surgical technique involves graft preparation, knee arthroscopy with removal of residual meniscus, posterior and anterior root fixation via independent transtibial tunnels, and circumferential fixation using a combination of all-inside and inside-out sutures.

The menisci have a crucial role in maintaining knee biomechanics, serving as load distributors and joint stabilizers, while providing proprioception to the knee.^1^ The treatment options for meniscal tears have evolved over time, and currently, we are seeing greater efforts at meniscal repair and preservation.^2^

Meniscal allograft transplantation (MAT) is indicated in young, symptomatic patients with meniscus deficiency, with limited chondral loss.^3^, ^4^, ^5^ The procedure aims to provide symptomatic relief and restore load transmission to potentially delay the onset of arthritis.^6^, ^7^, ^8^ Evidence suggests improvements in functional outcomes^9^, ^10^, ^11^ and possibly a chondroprotective effect.^7^^,^^12^ Furthermore, a recent systematic review reported 73.5% and 60.3% survivorship of allografts at 10 and 15 years posttransplantation.^13^

Several techniques for MAT have been described, including bony fixation and all-soft tissue fixation. The literature suggests bony fixation improves graft stability and results in less extrusion,^14^ in comparison to all-soft tissue fixation.^15^ However, no difference has been shown in clinical or functional outcomes or mid-term survivorship between these 2 different approaches.^16^^,^^17^ Graft-host size mismatch in MAT is common^18^ and can significantly reduce the effectiveness of the meniscus to reduce tibial plateau contact forces,^19^ leading to early graft failure.^11^

This surgical technique describes an arthroscopic MAT technique using a posterior root bone plug and anterior root all-soft tissue fixation. It is proposed that this approach combines the biomechanical advantages of posterior bone plug fixation with the technical benefits of all-soft tissue anterior root fixation with an adjustable loop to facilitate graft positioning and tension adjustment, enhancing graft fit and fixation (Table 1).

**Table 1:** Advantages and Disadvantages

Advantages	Disadvantages
▪ Allows greater adjustment of graft tension ▪ Can account for graft-host size mismatch ▪ Easier to find appropriate graft	▪ Capsule centralization requires an additional larger incision ▪ Increased cost of initial operation due to ACL TightRope (Arthrex) sutures

ACL, anterior cruciate ligament.

## Surgical Technique

This arthroscopic technique is shown on a left knee lateral meniscus (Video 1). However, it has been successfully applied to medial meniscus transplantation.

### Preoperative Evaluation

Indications for MAT include young and active patients with symptomatic meniscus deficiency following meniscectomy or trauma. Preoperative imaging includes weightbearing knee x-rays, long leg alignment x-rays, and magnetic resonance imaging to assess for meniscal deficiency, knee alignment, and concomitant ligamentous injury or chondral damage. Malalignment and instability must be corrected if identified. Meniscal allograft tissue is sourced from accredited tissue banks, with size matching using the Pollard method.^20^ We will typically accept a graft that is 1 to 2 mm larger than the templated size.^11^

##### Step 1: Patient Positioning and Preparation

The procedure is performed under a general anesthetic, with adductor canal and iPACK blocks. The patient is positioned supine, with the knee positioned to allow arthroscopic access in extension and at 90° of flexion. A thigh tourniquet is utilized sparingly.

##### Step 2: Meniscal Graft Preparation

Graft preparation is completed concurrently with step 3 if an experienced assistant is available.

**Fig 1:**
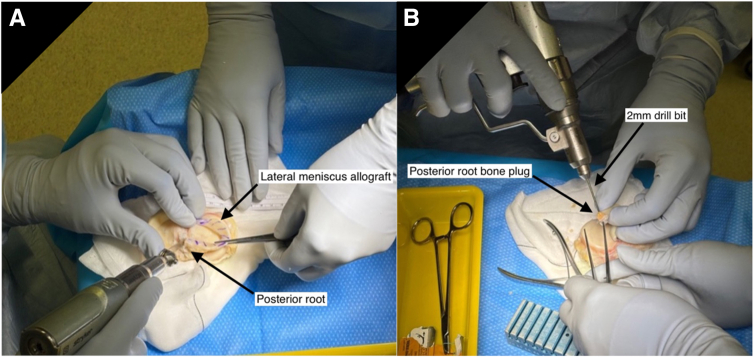
Preparation of the meniscal allograft is undertaken before arthroscopy on a side table under sterile conditions. It begins with the removal of the posterior meniscal root with an 8- to 10-mm diameter bone plug. An oscillating saw is used (A) to remove the posterior root of the allograft from the tibial plateau. After removal of the bone plug, a longitudinal hole is drilled through (B) with a 2-mm drill bit to facilitate insertion of the FiberLoop (Arthrex) sutures. Pictured: left knee meniscal allograft. No patient positioning or viewing portal in picture.

**Fig 2:**
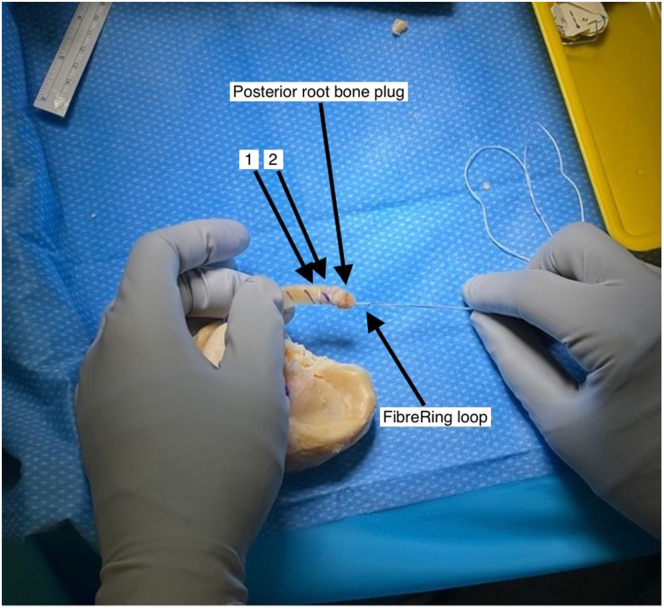
After creation of the posterior horn bone plug with a longitudinal hole drilled through, a FiberRing (Arthrex) suture is passed twice through the posterior horn at approximately 5-mm intervals (1) and (2) and then superiorly to inferiorly through the bone block. A free loop of FiberRing suture should be proud of the bone block for attachment of a TightRope (Arthrex). Photograph of left knee lateral meniscus allograft. No patient position or arthroscopy portal in figure.

**Fig 3:**
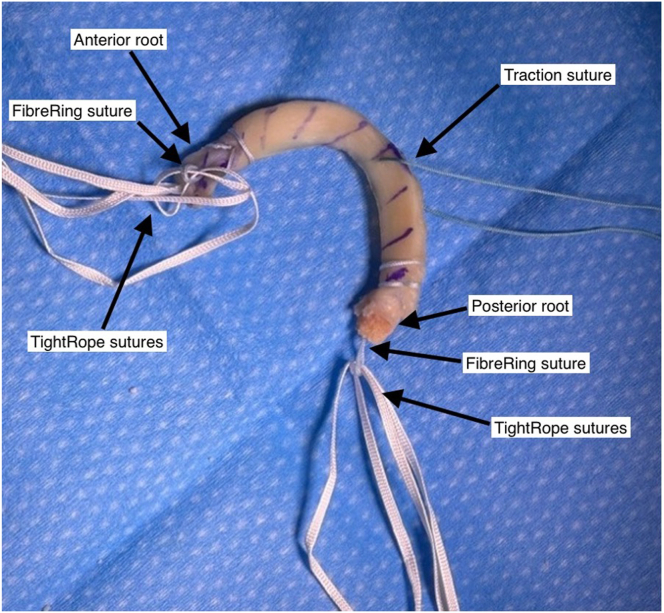
The meniscal allograft, after completing preparation, should appear as pictured. A TightRope (Arthrex) suture is fed through the free FiberRing (Arthrex) loop at both the anterior root and posterior root. A peripheral traction suture is positioned through the meniscal body at the junction with the posterior horn. Pictured: left knee lateral meniscal allograft. No patient position or arthroscopy portal in image

A cryopreserved, nonirradiated meniscal allograft is thawed in saline and washed in chlorhexidine. The posterior root is removed with a cylindrical bone plug measuring 8 to 10 mm in diameter and 8 mm in length using an oscillating saw and crimpers (Fig 1A). A 2-mm drill is used to create a longitudinal hole in the block (Fig 1B). A No. 2, 48-mm FiberRing (Arthrex) is passed twice through the posterior horn and then through the bone block (Fig 2). An ACL Repair TightRope (Arthrex) is attached to the free loop of suture on the underside of the bone plug (Fig 3). The anterior root is sharply dissected from the plateau, with an identical FiberRing and TightRope construct attached to the horn (Fig 3).

The meniscal body is prepared with a single traction suture at the junction of the posterior horn and body with a high-strength suture (Fig 3). The prepared graft is wrapped in vancomycin-soaked gauze (5 mg/mL) until implantation.

##### Step 3: Diagnostic Arthroscopy, Meniscal Evaluation, and Meniscectomy

**Fig 4:**
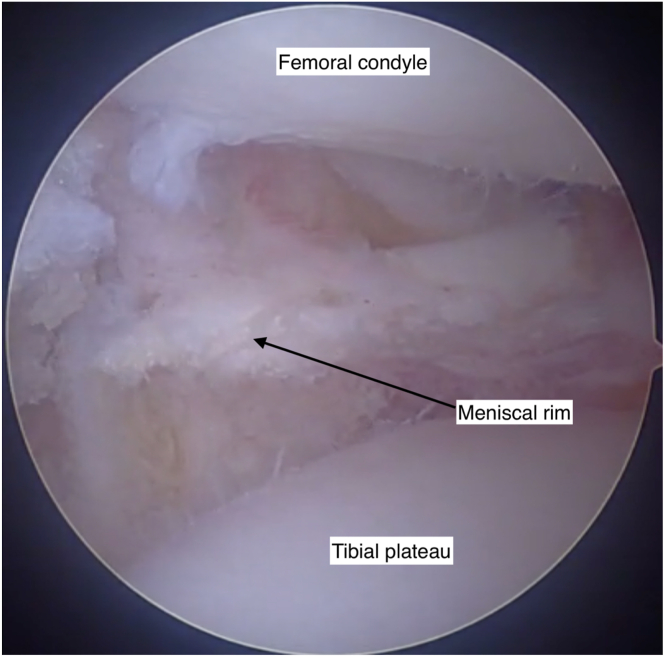
Debridement of the native meniscus is performed to prepare the knee joint for meniscal allograft transplantation. This arthroscopic view of the lateral joint capsule shows the post-debridement meniscus with a 1- to 2-mm meniscal rim retained to facilitate graft fixation and integration. View through an anteromedial arthroscopy portal into the lateral knee joint compartment. Knee flexed at 90 degrees.

Standard anteromedial and anterolateral arthroscopy portals are established. Diagnostic arthroscopy is performed and concomitant pathology addressed. Native meniscal remnants are resected to the meniscocapsular junction, preserving a peripheral rim of 1 to 2 mm to facilitate graft integration (Fig 4).

##### Step 3.5: Capsule Centralization

**Fig 5:**
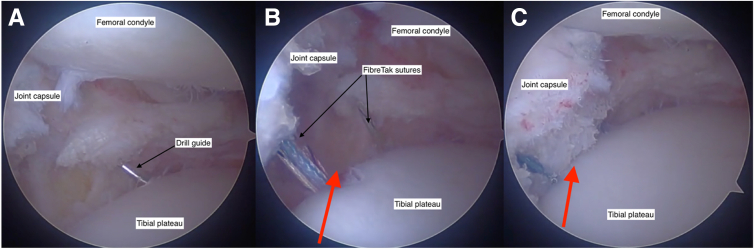
A capsule centralization procedure is supplementary to the meniscal allograft transplantation when diagnostic arthroscopy shows significant laxity between the joint capsule and the tibial plateau. A drill guide directs 2 transosseous FiberTak (Arthrex) suture anchors into the tibial plateau rim (A). The FiberTak sutures are then tightened outside the joint capsule to reduce the capsule to the tibial plateau. Space between the joint capsule and the tibial plateau is shown by the red arrow, before reduction (B) and after reduction (C). All three figures showing the view through an anteromedial arthroscopy portal into the lateral knee joint compartment. Knee flexed at 90 degrees.

Laxity of the meniscotibial ligament and localized capsule can predispose the MAT to extrusion.^7^ When present, we perform capsule centralization to supplement MAT. This adjunct procedure is performed through the same incision created for the inside-out sutures. Two Knotless FiberTak (Arthrex) anchors are drilled into the edge of the tibial plateau periphery (Fig 5A) with the sutures passed outside the capsule utilizing a suture lasso and tensioning into the other anchor. The centralization is tensioned before graft implantation (Fig 5).

##### Step 4: Posterior Root Tunnel Preparation

**Fig 6:**
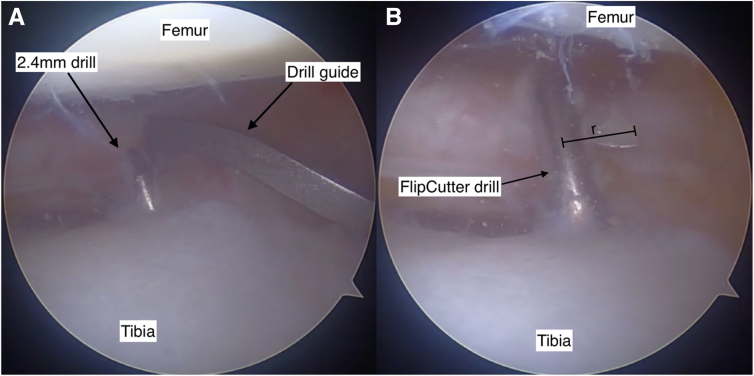
Arthroscopic view of the anatomic posterior root footprint during preparation of the posterior root tunnel (note camera is aiming medially). A drill guide is placed over the anatomic root footprint to direct a 2.4-mm predrill along the desired trajectory (A). The tunnel is overdrilled with a FlipCutter (Arthrex) (B), which is used to create a socket equal in size to the bone plug diameter. The blade length (r) determines the width of the socket. View through an anterolateral arthroscopy portal into the lateral knee joint compartment. Knee flexed at 90 degrees.

A root guide is positioned at the anatomic posterior root footprint and predrilled with a 2.4-mm pin (Fig 6A). This is overdrilled with a FlipCutter (Arthrex) with a socket size equal to the bone plug diameter (Fig 6B) and 2 to 4 mm deeper to allow further graft tensioning if required. A pulling suture is delivered through the tunnel into the knee.

##### Step 5: Graft Introduction and Positioning

**Fig 7:**
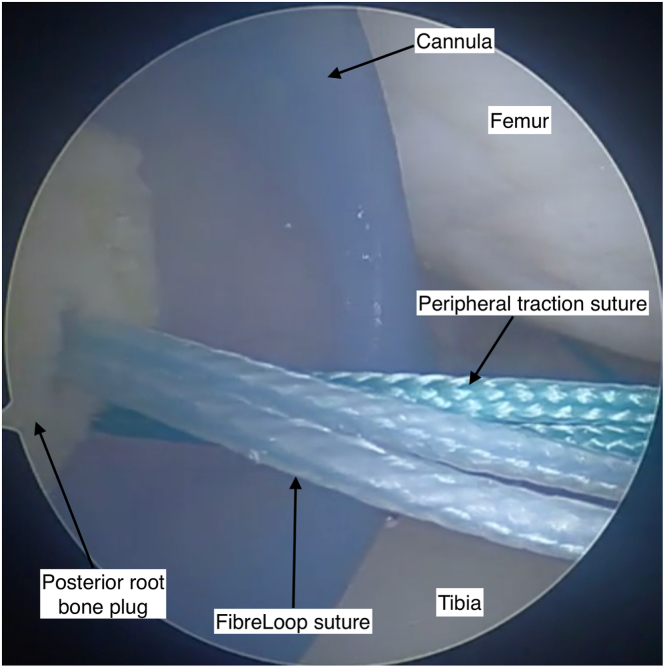
The introduction of the graft into the knee is facilitated by a PassPort Button Cannula (Arthrex) through an enlarged arthroscopy portal. The posterior root FiberLoop (Arthrex) suture is in view and has a TightRope (Arthrex) attached. The TightRope and the peripheral traction suture must be introduced first. The TightRope is used to guide the posterior root bone plug into position in the posterior root tunnel, and the peripheral traction suture guides the body of the meniscus into position. View through an anteromedial arthroscopy portal, viewing deep surface of the anterolateral knee arthroscopy portal. Knee flexed at 90 degrees.

**Fig 8:**
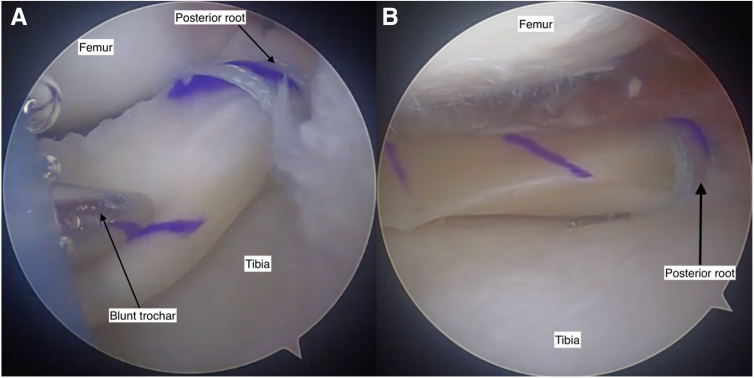
The meniscal allograft is gently pushed into the knee joint with a blunt trochar (A), ensuring constant tension on the posterior root TightRope (Arthrex) sutures to guide the bone plug into position, without bunching the sutures. When the graft is in position, the posterior root bone plug should no longer be visible (B), as it is seated in the posterior root tunnel. View through an anteromedial arthroscopy portal. Figure 8A is viewing deep surface of the anterolateral knee arthroscopy portal. Figure 8B is viewing the posterior foot of the lateral meniscus. Knee flexed at 90 degrees.

A small arthrotomy is created on the side of the transplant, and a PassPort Button Cannula (Arthrex) is inserted (Fig 7). A small posterolateral (or medial) incision is created, and dissection is performed to the capsule to allow inside-out suture retrieval. A single inside-out suture is passed to allow passage of the graft peripheral traction suture (Fig 7). The posterior root sutures are delivered down the tunnel. The graft is introduced through the cannula with continuous gentle tension applied to the posterior horn sutures while pushing the graft with a blunt trochar (Fig 8A), until the posterior horn bone plug is seated in the tunnel. The TightRope suture is provisionally tensioned, ensuring the bone block is well seated in the socket (Fig 8B). The peripheral traction suture is utilized to pull the body of the meniscus into position.

##### Step 6: Anterior Root All-Suture Fixation

**Fig 9:**
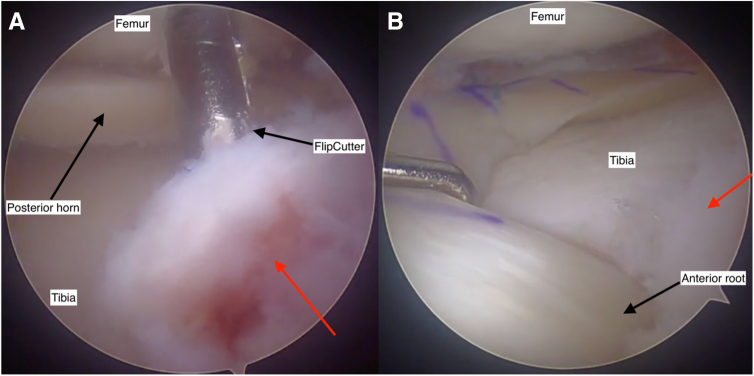
The anterior root tunnel is visualized during drilling using a 70° arthroscope. The FlipCutter (Arthrex) drill is seen entering the knee joint at the anatomic anterior meniscal root footprint (A) through the meniscal root remnant (red arrow). The anterior root TightRope sutures are then pulled through the anterior root tunnel to seat the anterior root, which can be seen disappearing into the tunnel (B). View through an anteromedial arthroscopy portal into the lateral knee joint compartment. Knee flexed at 90 degrees.

The anterior root tunnel is determined by the graft size (with a slightly nonanatomic position utilized if necessary for undersized grafts). A drill guide is positioned (usually the anterior cruciate ligament [ACL] guide is utilized), and the tunnel is predrilled with a 2.4-mm pin. A FlipCutter is then used to drill a 6-mm diameter socket of 10 mm depth (Fig 9A). The anterior root TightRope is passed down the anterior root tunnel. The graft is then preliminarily tensioned using the adjustable TightRope button (Fig 9B).

##### Step 7: Posterior Horn Fixation and Definitive Root Tensioning

**Fig 10:**
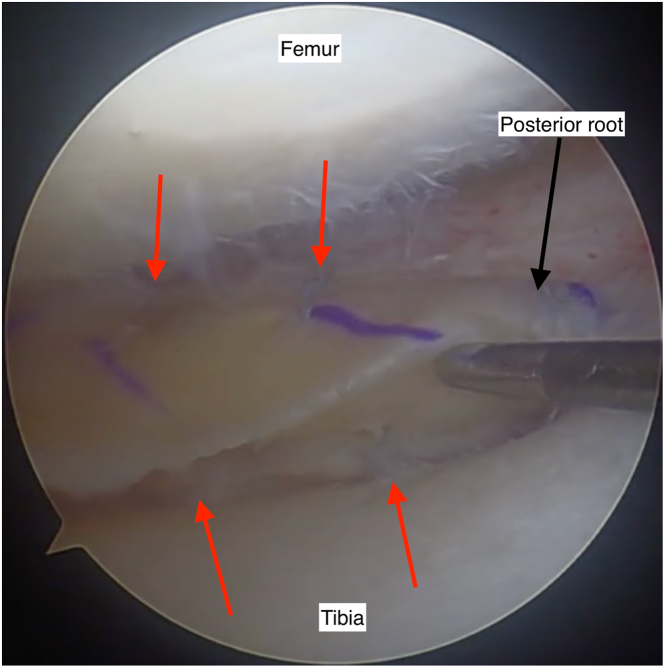
The posterior horn of the meniscal allograft is visualized with the posterior root bone plug seated. The all-inside posterior horn sutures are inserted as shown (red arrows), ensuring to spread the sutures on the superior and inferior surfaces of the meniscus. They are positioned at approximately 5- to 7-mm intervals, with care not to position them too close to the root attachment to reduce the risk of propagating a meniscal tear from the root. View through an anterolateral knee arthroscopy portal to view the lateral meniscus, posterior root.. Knee flexed at 90 degrees.

The graft position is assessed, and if required, more tension can be applied to the posterior root TightRope. The posterior horn is then fixed with 4 to 6 all-inside meniscus sutures, ensuring fixation is spread about 5 to 7 mm apart and on both superior and inferior sides of the meniscus (Fig 10). Tension should be maintained on the peripheral pulling suture during this procedure.

##### Step 8: Peripheral Fixation

**Fig 11:**
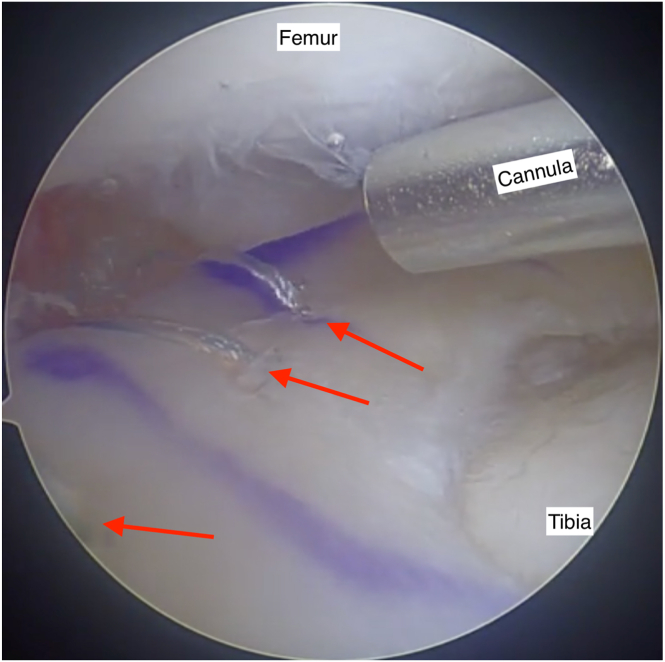
A section of the meniscal body is shown with 3 inside-out sutures (red arrows) placed along the periphery at approximately 5-mm intervals. These sutures are inserted using precontoured needles, depending on the region of the meniscal body intended for fixation. They are introduced through an Acufex cannula as shown. View through an anterolateral arthroscopy portal into the lateral knee joint compartment. Knee flexed at 90 degrees.

Peripheral fixation is achieved by placing inside-out sutures at 5-mm intervals above and below the meniscus using an Acufex cannula and precontoured meniscus suture and needles (Fig 11). The sutures are retrieved via the accessory incision and subsequently tied over the joint capsule.

##### Step 9: Final Graft Tensioning

The knee is cycled through a range of motion to assess graft positioning and tension. The anterior horn tension can be adjusted using the TightRope mechanism if required. Arthroscopic probing ensures the graft is well seated, with adequate apposition to the capsule. Once all sutures are tensioned, they are cut and closure is performed.

### Postoperative Care and Rehabilitation

Postoperatively, patients are restricted to nonweightbearing for 2 weeks, with a graded return to full weightbearing at 6 weeks. Early range of motion, muscle co-contractions, and isometric exercises are encouraged, with flexion restricted to 0° to 90° until 6 weeks.

Strength training and neuromuscular control exercises are introduced by week 6. A return to low-impact activities is allowed at 4 to 6 months, depending on achieving full range of motion and strength, proprioception, and endurance.

Most patients return to active lives, but high-impact sports are avoided.

## Discussion

MAT is a well-described surgical option for young, symptomatic patients. Modern implants and techniques can make the procedure less complex and possibly improve graft survival. This technique describes hybrid fixation to perform MAT using a posterior horn bone plug and anterior horn all-suture fixation with TightRope.

### Technical Considerations

For medial MAT, early release of the deep medial collateral ligament fibers improves visualization within the medial compartment to avoid iatrogenic chondral damage (Table 2). Additionally, shaving the medial tibial spine and removing synovium around the posterior cruciate ligament enhances visualization of the posterior root. Drilling the root sockets from the anterolateral tibia may result in a more anatomic position and easier drilling.

**Table 2:** Pearls and Pitfalls

Pearls	Pitfalls
▪ Early aggressive deep MCL release for medial meniscus ▪ Shave tibial spine and remove synovium from the PCL ▪ PUSH, don’t pull the graft in ▪ Consider osteotomy if there is malalignment after transplantation	▪ Beware debridement of ACL footprint with anterior root tunnel for lateral meniscus ▪ Minimize all-inside sutures within 5 mm of meniscal root ▪ Avoid overtightening of TightRope (Arthrex) sutures until after peripheral fixation

ACL, anterior cruciate ligament; MCL, medial collateral ligament; PCL, posterior cruciate ligament.

For lateral MAT, attention must be paid to the relationship between the anterior root and the ACL footprint, as the lateral anterior root insertion typically sits in close proximity to the ACL insertion (Table 2). Iatrogenic damage to the ACL may lead to instability and early graft failure. Consider a centralization of the distended lateral capsule in cases of chronic lateral meniscus deficiency.

A commonly reported zone of MAT graft failure is a radial tear adjacent to the root attachment. Due to this, we recommend minimizing the number of all-inside sutures within 5 mm of the root insertion, which may compromise the graft (Table 2). We have also started using the FiberRing for fixation of the roots as we believe this distributes the load upon the meniscus tissue more evenly in this high-load and poorly vascularized area. Since utilizing this technique, we have not seen any failures in this zone.

## Disclosures

The authors declare the following financial interests/personal relationships which may be considered as potential competing interests: F.T. has equity or stocks with Orthocell and receives speaking and lecture fees from 10.13039/100009026Smith & Nephew, Arthrex, and Device Technologies Australia Pty Ltd. All other authors (B.T., H.F.) declare that they have no known competing financial interests or personal relationships that could have appeared to influence the work reported in this paper.

## Supplementary Data

Video 1 This video shows the surgical technique for meniscal allograft transplantation with a hybrid posterior root bone plug and anterior root all-suture fixation. First, the steps for preparation of the meniscal allograft are shown with attachment of a TightRope (Arthrex) suture to both meniscal roots. Then the arthroscopic technique is revealed, showing step-by-step instructions for meniscal allograft insertion and fixation. The video is filmed during a meniscal allograft transplantation of a left knee lateral meniscus but has also successfully been applied to a medial meniscus. The arthroscopic procedure is conducted with the patient supine on a standard operating table with the knee positioned at 90° of flexion. The procedure is performed through anteromedial and anterolateral arthroscopy portals. First, the knee joint is prepared with debridement of the meniscal remnant. A supplementary capsule centralization is then shown. Next, the posterior root tunnel preparation is shown with introduction of the meniscal allograft into the knee. The anterior root tunnel preparation is shown and the anterior root seated in the tunnel. The posterior horn is fixed with all-inside sutures and the meniscus body with inside-out sutures. Graft tension is assessed throughout and can be adjusted at all stages.
